# A reference quality genome assembly for the jewel scarab *Chrysina gloriosa*

**DOI:** 10.1093/g3journal/jkae084

**Published:** 2024-04-17

**Authors:** Terrence Sylvester, Zachary Hoover, Carl E Hjelmen, Michelle M Jonika, Leslie T Blackmon, James M Alfieri, J Spencer Johnston, Sean Chien, Tahmineh Esfandani, Heath Blackmon

**Affiliations:** Department of Biology, Texas A&M University, College Station, TX 77843, USA; Department of Biology, University of Memphis, Memphis, TN 38111, USA; Department of Biology, Texas A&M University, College Station, TX 77843, USA; Department of Biochemistry and Biophysics, Texas A&M University, College Station, TX 77843, USA; Department of Biology, Texas A&M University, College Station, TX 77843, USA; Department of Biology, Utah Valley University, Orem, UT 84058, USA; Department of Biology, Texas A&M University, College Station, TX 77843, USA; Interdisciplinary Program in Genetics and Genomics, Texas A&M University, College Station, TX 77843, USA; Department of Biology, Texas A&M University, College Station, TX 77843, USA; Interdisciplinary Program in Ecology and Evolutionary Biology, Texas A&M University, College Station, TX 77843, USA; Department of Molecular Biosciences, University of Texas at Austin, Austin, TX 78712, USA; Department of Entomology, Texas A&M University, College Station, TX 77843, USA; Department of Biology, Texas A&M University, College Station, TX 77843, USA; Department of Biology, Texas A&M University, College Station, TX 77843, USA; Department of Biology, Texas A&M University, College Station, TX 77843, USA; Interdisciplinary Program in Genetics and Genomics, Texas A&M University, College Station, TX 77843, USA; Interdisciplinary Program in Ecology and Evolutionary Biology, Texas A&M University, College Station, TX 77843, USA

**Keywords:** Coleoptera genomes, sex chromosomes, sky islands, beetles

## Abstract

The jewel scarab *Chrysina gloriosa* is one of the most charismatic beetles in the United States and is found from the mountains of West Texas to the Southeastern Arizona sky islands. This species is highly sought by professional and amateur collectors worldwide due to its gleaming metallic coloration. However, the impact of the large-scale collection of this beetle on its populations is unknown, and there is a limited amount of genetic information available to make informed decisions about its conservation. As a first step, we present the genome of *C. gloriosa*, which we reconstructed using a single female specimen sampled from our ongoing effort to document population connectivity and the demographic history of this beetle. Using a combination of long-read sequencing and Omni-C data, we reconstructed the *C. gloriosa* genome at a near-chromosome level. Our genome assembly consisted of 454 scaffolds spanning 642 MB, with the 10 largest scaffolds capturing 98% of the genome. The scaffold N50 was 72 MB, and the BUSCO score was 95.5%. This genome assembly will be an essential tool to accelerate understanding *C. gloriosa* biology and help make informed decisions for the conservation of *Chrysina* and other species with similar distributions in this region. This genome assembly will further serve as a community resource for comparative genomic analysis.

## Introduction

The scarab beetle *Chrysina gloriosa* LeConte 1854 (previously known as *Plusiotis gloriosa*) is a charismatic beetle found in the continental United States and 1 of the 4 beetles in the genus *Chrysina* with a range that extends into the United States (LeConte 1854; Cazier 1951; Young 1957; Hawks 2001). Commonly known as the glorious beetle or glorious scarab, *C. gloriosa* has a metallic green body with silver stripes and blue eyes. Adult *C. gloriosa* depend on juniper trees as a food source, while larval forms depend on decaying logs (Ritcher 1966). In the United States, *C. gloriosa* is currently limited to higher elevation mountains from West Texas to the mountains of Southeastern Arizona, an area commonly known as the sky islands. High-elevation regions of these sky islands act as refugia for *C. gloriosa* and 3 other *Chrysina* species reaching the United States and are thought to represent remnants of a more widespread distribution that occurred during the Pleistocene epoch (Young 1957). These species of the genus *Chrysina* are a relic of the cooler and wetter era of the Pleistocene epoch (Young 1957).

Due to its colorful nature, *C. gloriosa* is highly sought by professional and amateur collectors worldwide (Genoways and Baker 1979). With the current trend of rising global temperatures and the aggressive collection of these beetles, extreme care may be justified to preserve the genetic diversity of these beetles in each mountain range. However, the absence of biological data sets and a lack of published biological literature of not only *C. gloriosa* but other beetles in this genus makes it difficult to make informed decisions about the conservation of these beetles. Much of the published literature on *Chrysina* species, including *C. gloriosa*, focuses on physical aspects of the coloration of the beetle, such as the polarizing properties of the cuticle (e.g. Sharma *et al*. 2009; Brady and Cummings 2010).

This paper presents the reconstructed genome of *C. gloriosa* as an initial step to aid in making informed conservation decisions for this species. Furthermore, our genome assembly will be the first chromosome-level assembly constructed for this charismatic beetle genus. A single female specimen collected in 2019 was used to perform Nanopore long-read sequencing for genome construction. In addition, Omni-C data were used to scaffold the genome at the chromosome level. The *C. gloriosa* genome assembly has a total length of 642 MB and a scaffold N50 of 72 MB. Our genome assembly will serve as a reference for mapping short-read data and variant discovery in an ongoing study of the population genetics of *C. gloriosa* and serve as a helpful resource in comparative genomic analysis.

## Methods

### Sample collection, DNA extraction, and sequencing

In an ongoing effort to document the population connectivity, *C. gloriosa* samples were collected from West Texas and Southeast Arizona during July and August from 2017 to 2019. We collected a total of 82 samples across 5 mountain ranges (Davis Mountains, Texas; Huachuca Mountains, Arizona; Chiricahua Mountains, Arizona; Madera Canyon, Arizona; and Piloncillo Mountains, Arizona). All specimens were collected at night between 7.00 Pm and 11.00 Pm (i.e. the first 4 h after sundown) using a combination of black light and Mercury vapor light. A single female specimen collected from Ida Canyon in the Huachuca Mountains (31.3807°N, −110.3298°W) on 2019 July 28 was used for genome construction.

To determine the biological sex of our specimen, the abdomen was dissected and examined for reproductive structures. DNA extraction and sequencing were done at the Texas A&M Institute for Genome Sciences and Society Core facility. Leg muscle tissue was dissected, and DNA was extracted using the Nanobind insect BIG DNA kit v 0.18 (Circulomics) following the Circulomics high-molecular-weight insect DNA extraction protocol. Extracted DNA integrity was assessed using a Genomic DNA ScreenTape on a TapeStation (Agilent). The Nanopore sequencing platform was used to generate long-read sequencing, and sequencing libraries were prepared following the manufacturer’s protocol using the SQK-RAD004 rapid sequencing library. Six R9.4.1 MinION flow cells generated 45.56 GB of sequencing data at an estimated 53× coverage (the estimated genome size [next section] was 850 MB using flow cytometry).

For all other 81 samples, a single muscle tissue was dissected from 1 hind leg of each beetle, and DNA extractions were performed using the QIAGEN blood and tissue DNA extraction kit (QIAGEN) following the manufacturer’s protocol. A NanoDrop One (Thermo Fisher) was used to examine the DNA extraction quality, while the Quantus Fluorometer (Promega) was used to quantify DNA. DNA was sequenced through the Texas A&M AgriLife Genomics and Bioinformatics Service center (https://www.txgen.tamu.edu/) using the Illumina short-read sequencing platform and 2 NovaSeq 6000 sequencing lanes. All specimens were sequenced using the 2 × 150 bp paired-end method at 1–2× coverage.

### Estimation of genome size

Flow cytometric methods following Johnston *et al*. (2019) were used to determine the *C. gloriosa* genome size. Neural tissue from individual frozen samples of *C. gloriosa* was dissected and deposited into 1 mL of Galbraith buffer. All samples were coprepared with a standard (lab stock of *Drosophila virilis*, genome size = 328 Mbp). Samples were gently ground with a Kontes “A” pestle ∼15 times to release nuclei. After passing samples through 41 mm mesh filters, samples were stained with 25 µL of 1 mg/µL propidium iodide and incubated in the dark. Samples were run on a Beckman Coulter CytoFlex flow cytometer with a 488 nm blue laser. Means of 2C nuclei fluorescence peaks were measured for both sample and standard using gating methods supplied within the instrument’s software before calculating the estimated genome size.

### Dovetail Omni-C library preparation and sequencing

Dovetail Genomics performed Omni-C library preparation and sequencing. For each Dovetail Omni-C library, chromatin was fixed in the nucleus with formaldehyde, digested with DNaseI, and extracted. The chromatin ends were repaired and ligated to a biotinylated bridge adapter, followed by proximity ligation of adapter-containing ends. After proximity ligation, crosslinks were reversed, and DNA was purified. Purified DNA was treated to remove biotin not internal to ligated fragments. Sequencing libraries were generated using NEBNext Ultra enzymes and Illumina-compatible adapters. Biotin-containing fragments were isolated using streptavidin beads before PCR enrichment of each library. The library was sequenced on an Illumina HiSeqX platform to produce ∼30× genome coverage.

### Genome assembly

Reads from each flow cell were concatenated into a single fastq file and reads below 1,000 bp were filtered using the program Filtlong v0.2.1 (https://github.com/rrwick/Filtlong). The genome was assembled using NextDenovo v2.5.0 (Hu *et al*. 2023) with default parameters setting the genome size to 850 MB based on the genome size estimate. Finally, the genome was polished using NextPolish v1.4.0 with default parameters (Hu *et al*. 2020).

Contaminant screening was performed using BlobTools v1.1.1 (Laetsch and Blaxter 2017). To generate the BlobTools map file, raw reads were mapped against the assembled genome using minimap2 v2.24 (Li 2018). The resulting sam file was converted to bam format using the samtools (v1.12) view module and sorted and indexed using samtools sort and index modules (Li *et al*. 2009; Danecek *et al*. 2021). To generate the BlobTools hits file, a preprocessed nucleotide BLAST database was downloaded from the National Center for Biotechnology Information (NCBI) (downloaded on 2021 October 25). The BLASTN tool of NCBI BLAST v2.12.0 was used to BLAST the contigs against the preformatted BLAST database to generate the hits file (Camacho *et al*. 2009). Using the map and hits file, BlobTools was used to create a blobplot showing the contamination level in the genome assembly.

### Assembly and annotation of mitochondria

The mitochondrial assembly of *Tribolium castaneum* was downloaded through the NCBI genome browser (accessed date 2022 May 25) and queried against the *C. gloriosa* assembly using NCBI BLAST v2.12.0 to filter mitochondrial contigs (Camacho *et al*. 2009). A dot plot was built using LAST v1045, comparing the *C. gloriosa* mitochondrial contig with the *T. castaneum* mitochondrial assembly for further confirmation. The LAST plots indicated that *C. gloriosa* mitochondria were assembled multiple times (back-to-back assembly). Therefore, *C. gloriosa* mitochondria were reassembled using a circularization tool preventing back-to-back assembly. We used contigs that completely covered the *T. castaneum* mitochondrial genome for the secondary assembly and circularization approach to avoid incorporation of nuclear mitochondrial DNA. All confirmed mitochondrial contigs were removed from the nuclear assembly.

All reads were mapped to the mitochondrial contig using minimap2 v2.24 and filtered aligned reads using the SAMtools view module (Li *et al*. 2009; Li 2018; Danecek *et al*. 2021). Spurious alignments were removed by filtering all mapped reads with mapping quality below 30. The resulting reads were assembled using Unicycler v0.4.9 under default settings to generate the mitochondrial assembly (Wick *et al*. 2017). The *C. gloriosa* mitochondrial contig was annotated using the MITOS 2 web server (Bernt *et al*. 2013) using RefSeq 89 Metazoa as the reference sequence and invertebrate as the genetic code. The maximum overlap parameter was set to 100, and “sensitive” was selected under the ncRNA tab. All other parameters were kept as default. Finally, the mitochondrial annotation was visualized using OG-DRAW v1.3.1 (Greiner *et al*. 2019).

### Scaffolding nuclear assembly

The initial contact map was created using Juicer v2.0 with the --assembly flag to generate input files necessary for 3d-DNA (Durand *et al*. 2016; Dudchenko *et al*. 2017). The scaffolding program 3d-DNA was run with 5 rounds of miss-join correction and used sites with a mapping quality of 30 or higher for scaffolding and visualization. Next, the assembly tools module in Juicebox v1.11 (JBAT) (Dudchenko *et al*. 2018) was used to curate the assembly output from 3d-DNA, following the recommendations of Howe *et al*. (2021). However, only large-scale, easily noticeable miss-assemblies (e.g. combining separate contigs into scaffolds) were curated. The software HiC-Hiker v1.0.0 (Nakabayashi and Morishita 2020) was used to further correct fine-scale misassembles present within scaffolds using a probabilistic approach to determine the possible orientation for a given set of contigs. The maximum distance for the probability of observing a contact between 2 loci (-K flag) was set to calculate automatically. Finally, the completeness of the *C. gloriosa* genome was assessed using BUSCO v5.2.2 (Simão *et al*. 2015). In addition, scaffolds were run separately as a query to a *T. castaneum* reference genome (Tcas5.2: GCF_000002335.3) and the scarab beetle *Trypoxylus dichotomus* genome (GCA_023509865.1) with minimap2 v2.24 (Li 2018). The resulting pairwise alignment file was used to generate Circos plots between the *C. gloriosa* and *T. castaneum* and *T. dichotomus* genomes using Circos v0.69-9 (Krzywinski *et al*. 2009).

### Sex chromosome identification

Both read depth and BLAST-based approaches were used to identify X chromosome scaffolds. For the read depth-based approach, filtered Illumina short reads from population-level sequencing were used. These short reads were filtered for adapter sequences and low-quality regions. Forward and reverse reads from each lane were combined to generate a single forward and a single reverse read file for each specimen. Reads were quality-checked using fastQC v0.11.9, and all reports were combined using multiQC v1.11 for analysis (Andrews 2010; Ewels *et al*. 2016). Then, fastp was used to remove adapter sequences and trim reads based on quality (Chen *et al*. 2018). Reads with a score of <20 on the Phred scale, and all unpaired reads were discarded, and only paired reads were used for mapping and sex chromosome identification.

The nuclear and mitochondrial genomes were combined into a single fasta file with both organelle and nuclear genomic information. The *C. gloriosa* genome was indexed using SAMtools faidx and BWA (v0.7.17) index modules (Li *et al*. 2009; Li and Durbin 2009; Li 2013; Danecek *et al*. 2021). All reads were mapped using the BWA-MEM algorithm, and the resulting SAM files were converted to BAM format using the SAMtools view module. Next, the mapped files were sorted by name and coordinate order using the SAMtools sort module. The SAMtools fixmate module was used to correct errors on read-pairing due to the alignment program, and the SAMtools markdup module was used to remove duplicate reads. Finally, mapped reads were extracted using the SAMtools view module. Only sites with a mapping quality >30 were kept to remove spurious mappings.

The mean read depth per scaffold was generated using the SAMtools depth command, and the mean depth of the 10 longest scaffolds was extracted, representing the 10 chromosomes of a typical scarab karyotype. In different specimens, the smallest of the 10 scaffolds had a similar or half the coverage compared with the rest of the examined scaffolds. Each specimen had varying coverage from 1× to 2× for the longest 9 scaffolds. Therefore, coverage across all 10 scaffolds was normalized to compare the coverage between specimens. For a given sample, the coverage of all scaffolds was divided by the mean coverage of the 9 longest scaffolds. From there, the coverage for scaffold 10 was compared with the rest of the scaffolds and assigned a biological sex based on the average coverage of scaffold 10.

To further validate our findings, the *C. gloriosa* scaffolds were queried against the *T. castaneum* genome and the matching scaffolds of *C. gloriosa* were compared with the *T. castaneum* X chromosome.

### Genome annotation

A de novo repeat library was created using RepeatModeler v2.0.4 to annotate the repeats in the genome assembly (Flynn *et al*. 2020). The species-specific de novo repeat library was combined with the Coleoptera-specific repeat library extracted from the RepeatMasker v4.1.5 repeat database (http://www.repeatmasker.org/RepeatMasker/). RepeatMasker v4.1.5 repeat database included a curated repeat library from Dfam v3.7 and RepBase v20181026 databases (Bao *et al*. 2015; Hubley *et al*. 2016). The utility script famdb.py was used to extract Coleoptera-specific repeat sequences. Then, RepeatMasker v4.1.5 was used with the custom repeat library to generate a soft-masked genome for genome annotation. We used the utility scripts calcDivergenceFromAlign.pl and createRepeatLandscape.pl of RepeatMasker v4.1.5 to calculate the amount of divergence within repeat sequence classes.

The software BRAKER v2.1.6 was used to annotate the genome using default parameters (Brůna *et al*. 2021) with no external evidence (ab initio) and with arthropod orthologous protein sequences as external evidence. The Arthropod protein sequences were downloaded from the orthologous sequence database OrthoDB v11 for genome annotation with protein sequence evidence, which included 4,307,558 sequences (Kuznetsov *et al*. 2023). To map the protein sequences to the soft-masked genome, ProtHint v2.6.0 was used with default parameters (Brůna *et al*. 2020). The resulting output of ProtHint was then used as the input for BRAKER, together with the soft-masked genome for annotation with protein sequence evidence.

The ab initio annotation and the protein sequence-based annotation were combined to generate the final gene transcripts using EVM v2.0.0 (Haas *et al*. 2008). ProtHint uses Spaln to generate a splice-aware genome annotation of protein sequences (Gotoh 2008). The annotation produced by Spaln was combined as protein sequence alignment for EVM. Finally, the functional domains of the final set of gene transcripts were annotated using InterProScan v5.60-92.0 (Jones *et al*. 2014). We assessed the quality of the annotation using BUSCO (endopterygota_odb10 data set) and web version of the OMArk (https://omark.omabrowser.org/home/). We generated annotation summary statistics using AGAT v1.2.1 (Dainat 2023).

## Results

### Assembly statistics

The *C. gloriosa* (Cglo_1.0: JAYRCI000000000) genome was built using Nanopore long-reads and scaffolded with Omni-C data. The initial assembly using Nanopore data yielded 239 contigs spanning 642 MB at a coverage of 44×, contig N50 of 8.7 MB, and the largest contig of 30.1 MB. The fact that our assembly size is smaller than the estimate based on flow cytometry suggests that some repetitive content was not assembled in the genome. The completeness of the assembly was assessed using BUSCO v5.2.2 and the endopterygota_odb10 data set as the core set of 2,124 single-copy orthologs genes. BUSCO results show that 95.8% of the single-copy orthologs are present in the assembled genome (94.6% complete single copy, 1.2% complete duplicated, 2.9% fragmented, and 1.3% missing). Our contamination screening pipeline identified a single contig matched with a prokaryotic species. This contig was removed from the genome, and the new filtered genome was used for subsequent assembly processes.

**Fig. 1.**
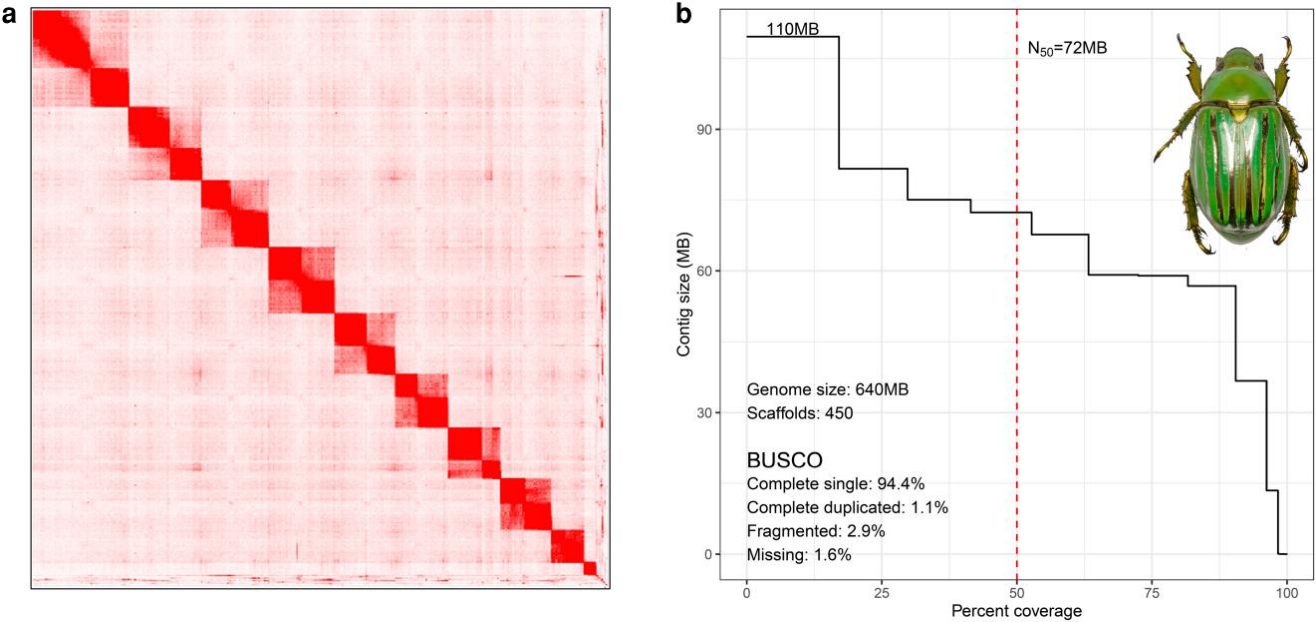
a) HiC contact map of *C. gloriosa.* b) Assembly statistics and completeness of the *C. gloriosa* genome (genome size is 640 MB, the longest scaffold is 110 MB, scaffoldN50 is 72 MB, and GC composition is 35.9%). In BUSCO analysis, 95.5% of the orthologs are complete single or duplicated, and 3.5% are fragmented or missing.

The genome scaffolding pipeline initially identified 492 scaffolds, with 19 scaffolds exceeding 1 MB in size. Manual curation using JBAT placed these 19 scaffolds into 10 scaffolds representing the 10 chromosomes in a typical scarab beetle karyotype (Fig. 1a). Smaller scaffolds were also arranged when the connections were clear. This process reduced the total number of scaffolds to 454. The final assembly had a scaffold N50 of 72 MB, and the largest fragment size was 109 MB (before curation, the N50 was 37 MB, and the largest fragment size was 75 MB). The largest 10 scaffolds covered 98.3% of the genome. When assessed for the completeness of the scaffolded assembly using BUSCO v5.2.2, we did not observe a significant change in the BUSCO scores when compared with the contig level assembly (94.4% complete single copy, 1.1% complete duplicated, 2.9% fragmented, and 1.6% missing) (Fig. 1b).

### Repetitive sequence and genome annotation

**Fig. 2.**
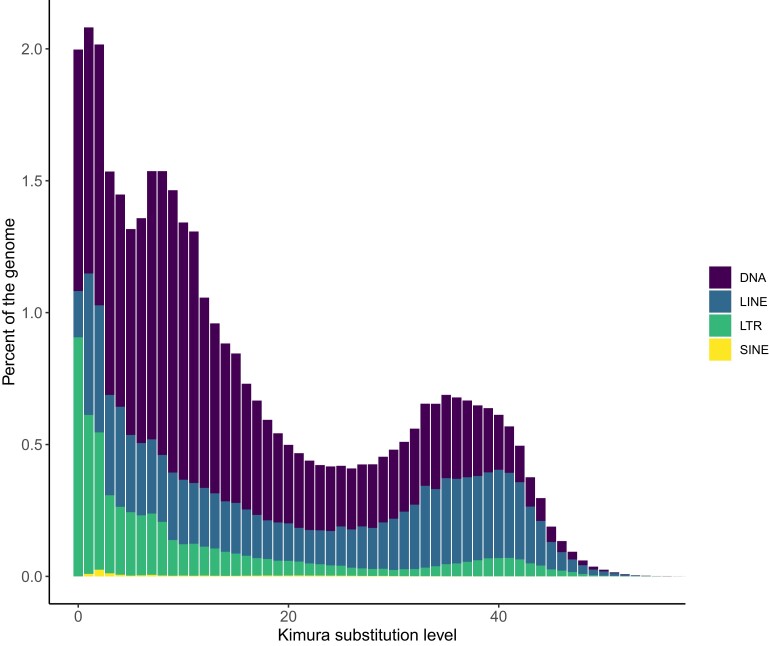
Kimura substitution level of the repetitive sequences identified in the *C. gloriosa* genome.

Annotation of repeats identified 52.94% of the genome as repetitive sequence. Most repeats were DNA transposons (20.58% of the genome), followed by retro elements (15.60% of the genome), and satellites and simple sequence repeats (0.70% of the genome). Accumulation of repetitive sequences along the scaffolds shows enrichment of repetitive elements toward the center of the chromosomes, indicating the centromeric regions (Supplementary Fig. 1). The divergence of repetitive sequences was assessed, and 3 distinct peaks were suggestive of 2 separate repetitive sequence expansion events in the past (one more recent and one in the distant past) (Fig. 2).

The genome annotation pipeline identified 19,421 gene transcripts (Supplementary Table 1). BUSCO analysis using the endopterygota_odb10 core gene set identified 77.5% of the single-copy orthologs are present in the genome annotation (76.1% complete single copy, 1.4% complete duplicated, 7.6% fragmented, and 14.9% missing). Assembly completeness using OMArk identified 86.55% of the conserved hierarchical orthogroups (4,840 at the level of Endopterygota) are present indicating that we have captured a large portion of the gene space in our structural annotation (Supplementary Fig. 2). Functional assignment using InterProScan assigned 16,257 gene transcripts with functional domains.

### Genome synteny

**Fig. 3.**
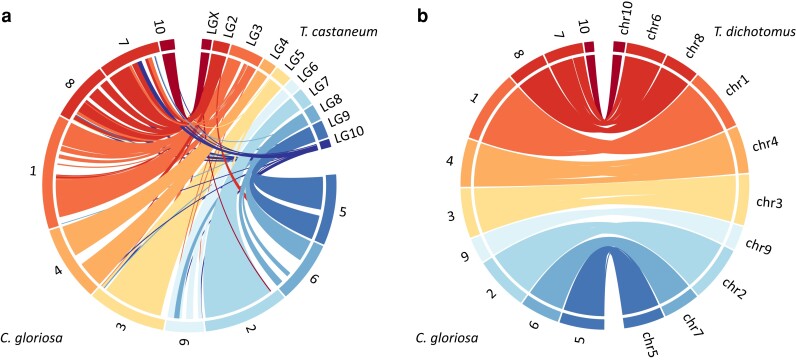
Circos plots comparing the *C. gloriosa* genome with the (a) *T. castaneum* genome and the (b) *T. dichotomus* genome.

The *C. gloriosa* assembly was compared with *T. castaneum* and *T. dichotumus genomes*. The comparison of *T. castaneum* indicates that linkage group (LG) 2 of *T. castaneum* shows synteny with 2 scaffolds of *C. gloriosa* (Fig. 3a). Furthermore, LG10 of *T. castaneum* matches several scaffolds in *C. gloriosa*. The comparison with *T. dichotumus* indicates that each of the *C. gloriosa* scaffolds has a clear 1-to-1 orthology with scaffolds in the *T. dichotumus* assembly (Fig. 3a). Despite this structural conservation, frequent inversions are documented within chromosomes. The LGX (the X chromosome) of *T. castaneum* has a clear orthology with scaffold 10 of *C. gloriosa*, providing potential evidence for the X chromosome scaffold. Mapping short-read sequences back to the genome assembly shows that all scaffolds except for scaffold 10 have similar coverage across all specimens (Supplementary Fig. 3). Some specimens have a normalized coverage near 1×, while others have a coverage near 0.5×. This further confirms that scaffold 10 is the X chromosome of *C. gloriosa*.

### Assembly and annotation of the mitochondrial genome

The mitochondrial genome was assembled using Unicycler and annotated using the MITOS 2 web server. The *C. gloriosa* mitochondrial genome assembly was 17,644 bases in size and consisted of 13 protein-coding genes, 2 ribosomal genes, and 22 tRNA-coding genes (Supplementary Fig. 4).

## Discussion

*C. gloriosa* is 1 of only 4 *Chrysina* species that have a distribution reaching the United States. Until now, no whole-genome data for a *Chrysina* species in the United States existed, making it difficult to understand their evolution and assess the need to conserve these charismatic beetles. As a first step in understanding the biology and evolution of this beetle, a chromosome-scale genome assembly for *C. gloriosa* was built using a combination of long-read sequencing and Omni-C data. The genome assembly consists of 10 large scaffolds capturing 98.3% of the genome, representing a typical Scarabaeidae 10 chromosomes (9 autosome pairs and an XY chromosome pair). The X chromosome scaffold of the *C. gloriosa* genome assembly was identified through synteny analysis with the *T. castaneum* genome and further validation using individual read-depth data.

To our knowledge, this is the only genome assembled to chromosome-level for the genus *Chrysina*. The only other genome assembly available in this genus is for *C. resplendens* which used short-read sequencing data. The *C. gloriosa* assembly size is slightly larger at 642 MB than *C. resplendens* assembly, which has a size of 611 MB spread across 113,068 scaffolds. However, these species have smaller genomes than *Chrysina woodii* (another *Chrysina* species that reaches the United States), whose genome size is estimated to be 856 MB using flow cytometry (Hanrahan and Johnston 2011). Compared with the *C. resplendens* genome, the completeness of our genome is relatively high, with 94.4% of the BUSCO genes present as complete single copies. In contrast, the *C. resplendens* genome has a BUSCO single-copy score of just 68.6% (Feron and Waterhouse 2022).

In summary, the *C. gloriosa* genome assembly is the first step in a long-term project to monitor population connectivity and demography of *Chrysina* species in the sky islands of the southwestern United States. This genome will serve as a starting point to answer fundamental questions about the resilience and connectivity of populations and help to determine the need to conserve this species in the future. Finally, this assembly will serve as a community resource in understanding the evolutionary dynamics of genomes in Scaraboidea and Coleoptera.

## Supplementary Material

jkae084_Supplementary_Data

## Data Availability

Sequencing data are available at NCBI GenBank under the BioProject PRJNA1043134. Other data, results, and scripts used for data processing and generating figures are available at the following GitHub repository: https://github.com/Tsylvester8/Cglo-genome. Supplemental material available at G3 online.
