# Supplementary material for: A reference quality genome assembly for the jewel scarab Chrysina gloriosa
Source: G3 (Bethesda). 2024 Apr 17;14(6):jkae084. doi: 10.1093/g3journal/jkae084 (PMC11152064; doi:10.1093/g3journal/jkae084)
Supplement: jkae084_Supplementary_Data [file jkae084_supplementary_data.docx]

**Supplementary figures and tables**

**
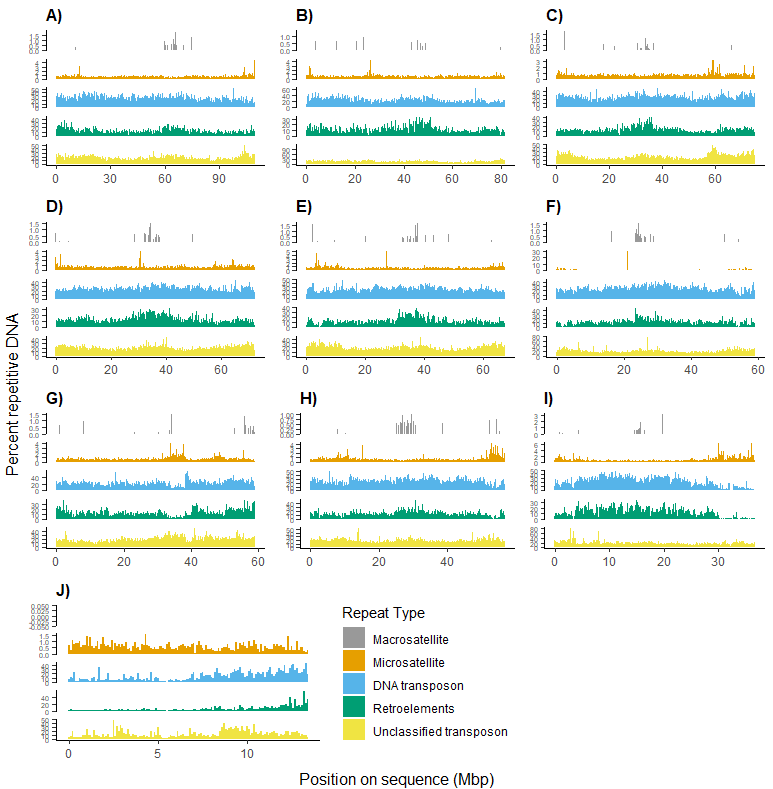
**

**Figure S1** Distribution of repetitive content across the ten largest scaffolds in the *Chrysina gloriosa* genome. Repetitive content was classified into five categories: (from top to bottom of each panel) macro and micro-satellites, DNA transposons, retrotransposons, and unclassified transposons. Panels A through J represent scaffolds 1 through 10, respectively.


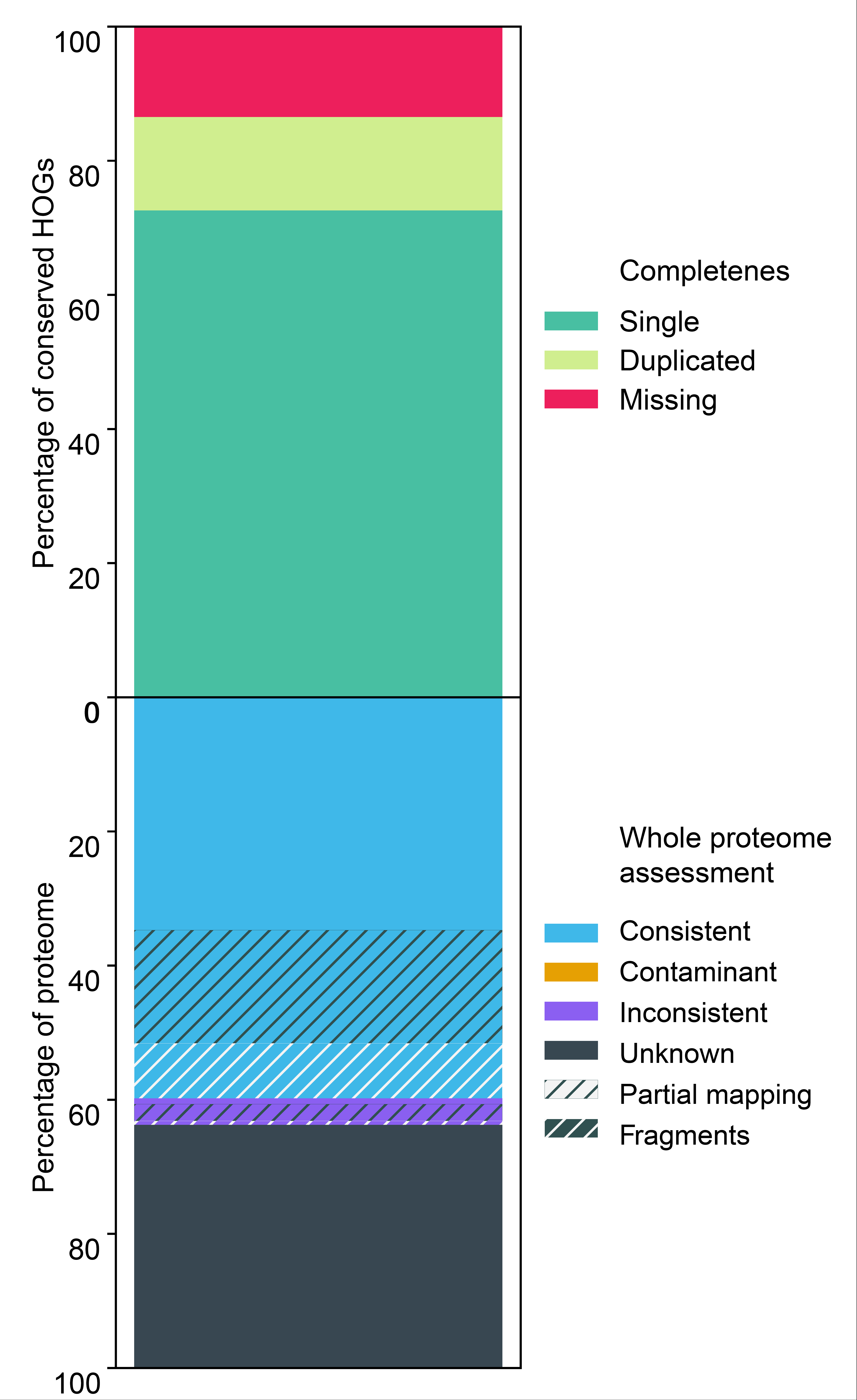


**Figure S2** Proteome quality assessment using OMArk with Endopterygota hierarchical orthogroups (HOGs) as the core gene set (4840 HOGs). Assessment of completeness shows 13.45% of HOGs as missing; 13.95% duplicated (with 2 genes (0.04%) showing unexpected duplications); 72.60% as single. Consistency assessment (whole proteome assessment) shows that 11615 genes were placed in the selected lineage (Endopterygota). There were 767 genes that placed elsewhere, and 7039 genes did not have close homologs in the selected lineage and had unknown lineage placement.

**
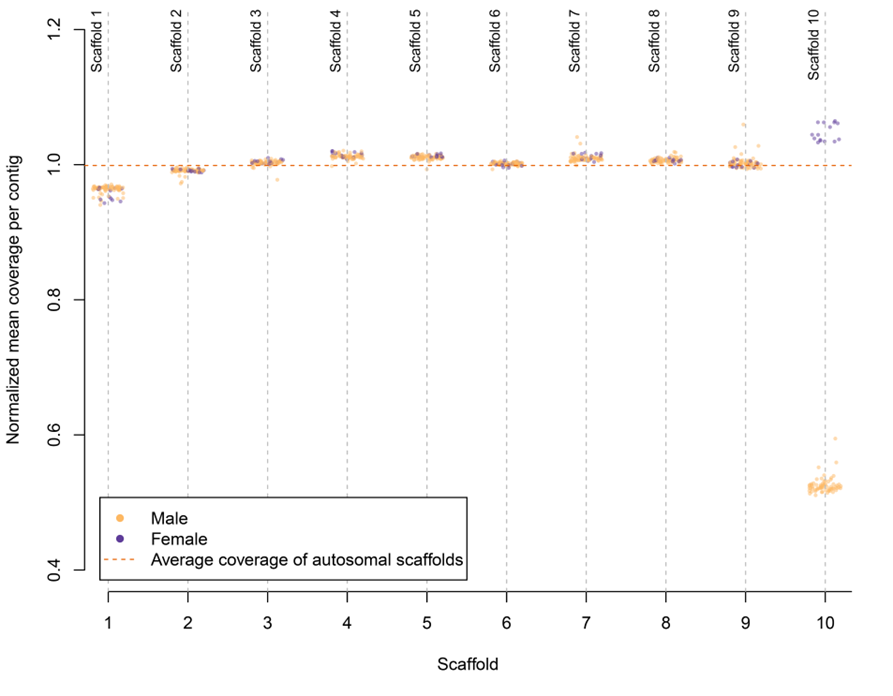
**

**Figure S3** Distribution of the normalized mean coverage of reads from each sample mapped back to the ten largest scaffolds of the *Chrysina gloriosa* genome. Scaffolds are organized in decreasing size. All except scaffold ten has nearly 1x coverage for all specimens, while scaffold 10 has about 1x coverage for some specimens and close to 0.5x coverage for others. The biological sex of each sample is assigned based on the coverage distribution at Scaffold 10.


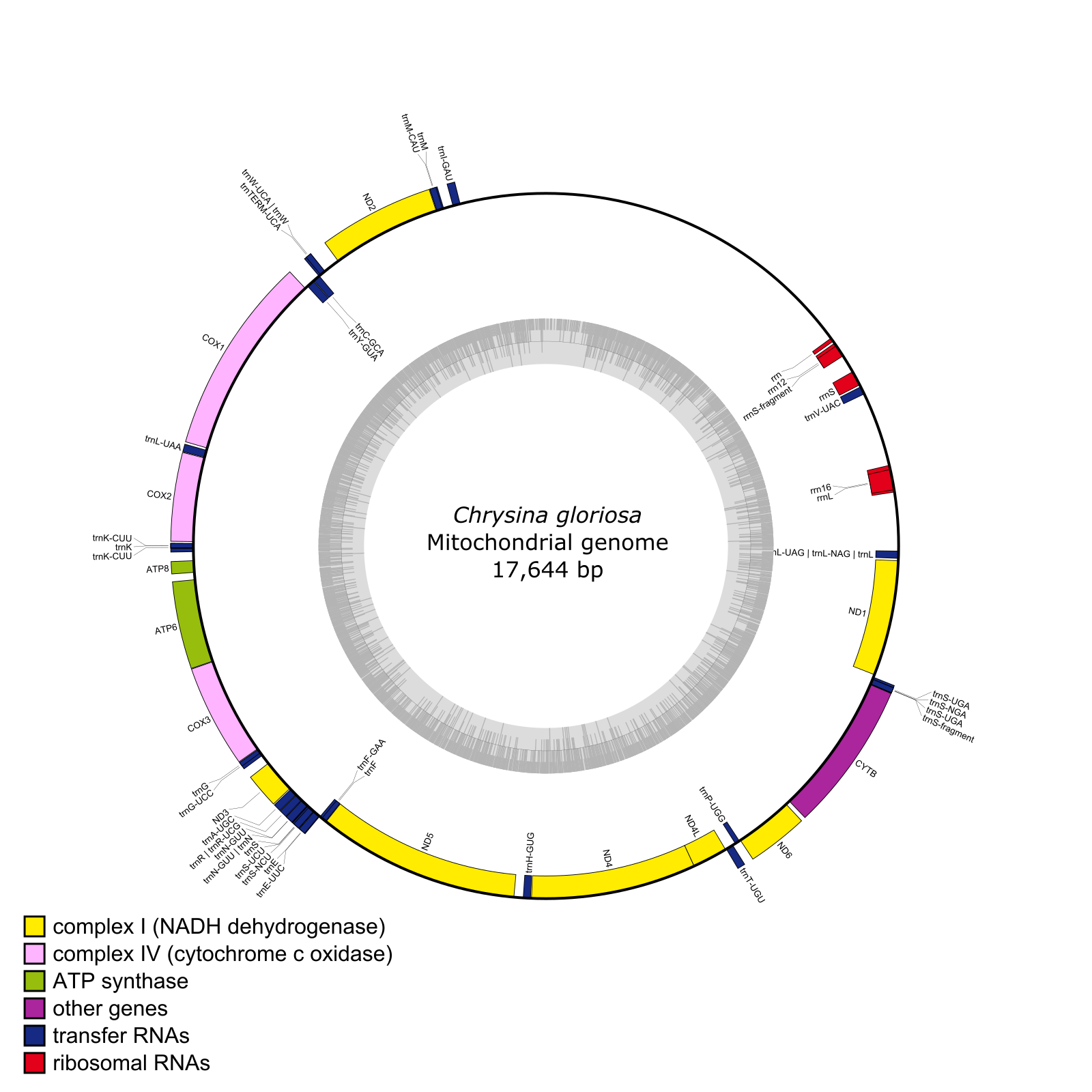


**Figure S4** Circular map of the *Chrysina gloriosa* mitochondrial genome. The inner ring is the GC percentage, and the outer ring is the mitochondrial genome's annotated features. Each feature complex (e.g., cytochrome oxidase subunits, ATP synthase proteins, tRNAs) is assigned a unique color.

**Table S1** Summary statistics of the *Chrysina gloriosa* genome annotation. Here we used cds to denote the complete collection of exons.

| **Statistic** | **Value** |
| --- | --- |
| Number of genes (mRNAs) | 19421 |
| Number of exons | 80818 |
| Number of introns | 61397 |
| Number overlapping genes | 0 |
| Number of single exon genes | 5120 |
| mean exons per gene | 4.2 |
| mean introns per gene | 3.2 |
| Total gene length (bp) | 115386500 |
| Total exon length (bp) | 21070724 |
| Total intron length (bp) | 94315776 |
| mean gene length including all features (bp) | 5941 |
| mean cds length (bp) | 1084 |
| mean exon length (bp) | 260 |
| mean intron length (bp) | 1536 |
| % of genome covered by genes | 18 |
| % of genome covered by cds | 3.3 |
| % of genome covered by introns | 14.7 |
| Longest gene (bp) | 128956 |
| Longest exon (bp) | 14647 |
| Longest intron (bp) | 42463 |
| Shortest gene (bp) | 151 |
| Shortest exon (bp) | 3 |
| Shortest intron (bp) | 29 |
